# Evaluating clinical decision support software (CDSS): challenges for robust evidence generation

**DOI:** 10.1017/S0266462324000059

**Published:** 2024-02-08

**Authors:** Mah Laka, Drew Carter, Tracy Merlin

**Affiliations:** Adelaide Health Technology Assessment (AHTA), School of Public Health, University of Adelaide, Adelaide, SA, Australia

**Keywords:** Clinical decision support systems, CDSS, quality of health care, digital health, evaluation, assessment, Real world evidence

## Abstract

**Objectives:**

Computerized clinical decision support software (CDSS) are digital health technologies that have been traditionally categorized as medical devices. However, the evaluation frameworks for traditional medical devices are not well adapted to assess the value and safety of CDSS. In this study, we identified a range of challenges associated with CDSS evaluation as a medical device and investigated whether and how CDSS are evaluated in Australia.

**Methods:**

Using a qualitative approach, we interviewed 11 professionals involved in the implementation and evaluation of digital health technologies at national and regional levels. Data were thematically analyzed using both data-driven (inductive) and theory-based (deductive) approaches.

**Results:**

Our results suggest that current CDSS evaluations have an overly narrow perspective on the risks and benefits of CDSS due to an inability to capture the impact of the technology on the sociotechnical environment. By adopting a static view of the CDSS, these evaluation frameworks are unable to discern how rapidly evolving technologies and a dynamic clinical environment can impact CDSS performance. After software upgrades, CDSS can transition from providing information to specifying diagnoses and treatments. Therefore, it is not clear how CDSS can be monitored continuously when changes in the software can directly affect patient safety.

**Conclusion:**

Our findings emphasize the importance of taking a living health technology assessment approach to the evaluation of digital health technologies that evolve rapidly. There is a role for observational (real-world) evidence to understand the impact of changes to the technology and the sociotechnical environment on CDSS performance.

## Introduction

Clinical decision support software (CDSS) refers to a class of medical software designed to improve the quality and safety of care by supporting evidence-based clinical decision making (1). CDSS combines patient information with a targeted clinical knowledge base to provide treatment recommendations and assessments specific to each patient at the point of care (2). Traditionally, CDSS was designed to be knowledge-based, in that system outputs were based on data from the medical literature and then appropriately targeted using conditional statements. With evolving technology, a newer class of non-knowledge-based CDSS has emerged using artificial intelligence (AI) and machine learning (ML) to provide clinical recommendations (3).

The need for innovative systems like CDSS is driven by many challenges in health care. These include the high volume of data that needs to be analyzed in real-time for clinical decision making and the need to provide personalized care. A successful integration of CDSS into the care process provides various benefits (4). CDSS can reduce medication errors, provide timely reminders for medical surveillance, increase practitioner adherence to clinical guidelines, promote cost-effective treatments, and improve diagnostic capabilities (5).

However, studies have emphasized that an effective evaluation is required to establish the clinical validity of the CDSS system, but also to ensure that recommendations provided by the system are functionally meaningful, up-to-date, and fitted to the clinical context (6;7). An evaluation can also help to avoid a range of legal, ethical, and clinical problems associated with the use of CDSS in clinical decision making. For instance, CDSS increases the risk of “automation bias,” whereby end-users overly rely on the software’s recommendations without considering the specific clinical context. This could have consequences for patient care if the advice provided by the software is wrong. Similarly, the risk of introducing prescribing errors increases if the clinical knowledge base embedded in the software is not up to date (8). This might be more pertinent in non-knowledge-based CDSS because the algorithms used are opaque (a “black-box”). Thus, users are not aware of how the software came up with specific recommendations, and whether the software appropriately factored in patient specifics.

The lack of comprehensive evaluation models in digital health has been attributed to the misalignment between fast-paced technological innovation and restrained and time-consuming change in healthcare systems (9;10). The pace of development in the healthcare industry is typically regulated by demand and stringent regulatory requirements that essentially result in a slower growth cycle (11). However, digital health systems, such as CDSS, with their rapid development and iterative upgrades, do not conform to this paradigm. We aimed to address the gaps in evaluation methodologies and knowledge by identifying the potential strengths and weaknesses of existing evaluation approaches for CDSS. Specifically, we posed the following research question: *What are the challenges for robust and timely evaluation of CDSS to ensure the quality and safety of care*?

Most evaluation studies have focused on ensuring that CDSS meets end-user requirements, whereas only a limited number of products have been subjected to stringent pre- and post-market evaluation (12). In this study, we examine the gaps and challenges for CDSS evaluation across different phases of the product life-cycle, from pre-market evaluation to post-market monitoring. Pre-market evaluation includes regulatory market approval to ensure that the digital system demonstrates safety and effectiveness. After market approval is granted, post-market assessment may consist of health technology assessment (HTA) for funding/reimbursement purposes, performance evaluation within the implementing organization against expected outcomes, and monitoring by regulatory bodies of the software’s safety and performance.

## Methods

### Study Design

We adopted a qualitative approach to try to gain an in-depth understanding of the phenomenon of CDSS evaluation, where there are many unknowns. We carried out semi-structured interviews to explore different aspects of CDSS evaluation and to generate knowledge on different gaps in the evaluation process. The interview schedule was informed by our previous study involving a systematic review of the CDSS literature (13), plus a range of other systematic reviews (2;6;10–12;14). The current study specifically focuses on a subset of questions related to the evaluation of CDSS (see Supplementary Material). The study was approved by the University of Adelaide’s Human Research Ethics Committee (approval number: H-2019-094).

### Recruitment and Data Collection

The recruitment and data collection strategy has been detailed elsewhere (15). The participants were recruited from organizations involved in digital health innovations in the Australian healthcare system. Invited participants were interviewed only if they had been involved in the implementation, evaluation, or regulation of digital health systems such as CDSS.

Verbal and written consent were obtained before conducting the interviews. The interviews were conducted by the first author (ML) using the interview guide provided in the Supplementary Material. The interview questions were open-ended to elicit detailed discussion on participants’ experiences of evaluating CDSS, the challenges they faced and recommendations for improving the process. The questions specifically focused on understanding CDSS evaluation in Australia and its merits and shortcomings in considering organizational and clinical contexts, stakeholder perspectives, and the nature of evolving technology. Data collection was terminated after 11 interviews due to data saturation, with no new themes being raised in the final two interviews. All interviews were audio-recorded and transcribed verbatim.

### Theoretical Framing

CDSS are sociotechnical systems characterized by the interaction of humans, technology, and health systems. Therefore, the evaluation of CDSS must not be limited to a usability assessment but also encompass how well CDSS integrate into the broader healthcare system. This requires answering *what, how, who, when,* and *why* questions in the evaluation process. Many evaluation frameworks adopted from information systems literature are unable to consider the contextual and cultural sensitivities of the healthcare setting (9;16).

Some previous evaluation studies have adopted a Content, Context, and Process (CCP) framework (10;12;16). This framework was originally developed to study organizational changes, but several researchers later adopted it in health information systems (HIS) evaluation studies. It provides a holistic approach to evaluation by extending the assessment from technical factors to multiple contextual factors that influence CDSS performance.

**Figure 1.**
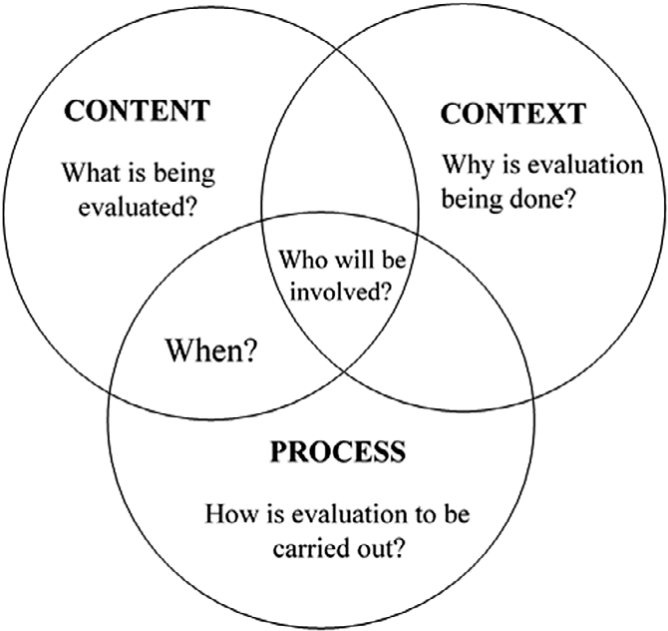
The content, context, and process (CCP) theoretical framework.

The data analysis in this study was underpinned by the CCP framework (Figure 1), as adapted by Stockdale and Standing (16).

In this framework, “content” is associated with understanding precisely what needs to be evaluated. Stockdale and Standing argue that the use of a socio-technical paradigm broadens the scope of evaluation from quantifiable benefits such as cost savings to varying opportunities and risks presented by HIS.

Although evaluation is a complex process, content or “what needs to be measured” is determined by the context of the organization and stakeholder requirements. “Context” refers to why an evaluation must be carried out and who needs to be involved. Sockolow et al. (17) indicate that context has been unduly neglected in HIS evaluation, with aspects such as organizational, political, and professional considerations usually missing in evaluation studies.

The “process” aspect of the CCP framework focuses on how and when evaluation should be carried out. It encompasses assessing the sequence of activities, individuals, and events related to the implemented system.

### Data Analysis

First, the transcripts were read repeatedly to enhance understanding and familiarity with the data. Using NVivo 12, data were then coded in both a data-driven, inductive way and a theory-based, deductive way, with the theory being the above-detailed CCP framework. The method of inductive analysis proposed by Boyatzis (18) helped in the identification of *a posteriori* codes reflecting important patterns of meaning observed in the data. The inductive coding was semantic in that it was directed by the explicit content of the interview transcripts. The first interview was independently coded in this way by two researchers (ML and DC), who compared codes and discussed differences, agreeing on the way forward. Along with the inductive analysis, we also used the deductive approach provided by Crabtree and Miller, creating *a priori* codes based on the CCP theoretical framework and the research question (19). The different aspects (Content, Context, and Process) and underlying questions (why, what, who, how, and when) of the CCP framework were used to organize data during the analysis. Finally, codes were refined and collated into broader themes that helped us to understand different aspects of the underlying phenomenon. For example, participants identified that evaluation requirements may vary for different stakeholders depending on their specific context and objectives, though participants also believed that consensus is required among stakeholders to establish a shared baseline standard for CDSS quality. These concepts were separately coded as “stakeholders’ priorities” and “baseline standards.” As analysis progressed, it became apparent that both codes related to the overarching concept of “what” should be measured, or selecting the right indicators of CDSS success.

## Results

Our results show that planning CDSS evaluation as a mere technical process rather than understanding it in a dynamic socio-technical context has limited the ability to sufficiently recognize the benefits and risks associated with CDSS implementation. We present our results under four themes, which correspond to different questions concerning evaluation activities.

### Why Evaluate?

Participants stated that due to the complexity of the care environment, comprising interrelated processes where change in one element impacts others, evaluation is critical in assessing the interaction of CDSS with a health system, organizations, and end users.

Many participants believed that the implementation and use of CDSS are embedded in a sociotechnical context characterized by human behavior, culture, and politics. For this reason, many evaluation models for digital health systems, developed from a business context, are not suitable for healthcare settings. Participants indicated that this was reflected in many CDSS evaluation processes they have witnessed in Australia, which isolate the system performance from the interdependent processes such as workflow, human interaction with the technology, and organizational practices In many cases, the evaluation is an afterthought, therefore it lacks the specificity required for assessing system effectiveness.

if you don’t have a proper evaluation for going back to see how your care services and the clinical process has improved after the implementation of decision support tools, then what the system ends up doing is being a subject to some business goals and political drives for addressing a specific problem which may not actually be the main concern for patient care. (Participant 09)

In this regard, several participants felt that current evaluation processes tend to focus on system accuracy (e.g., did the software produce the correct prescribing recommendation?) while overlooking the system impact on process outcomes and end-user performance (e.g., did the systems’ recommendations help in improving clinical-decision making and did it impact the quality of care?).

[The] design team who are very focused and knowledgeable and they have a good clinical input, they predict that product can reduce the medication misadventure by 25 percent, and then you go to deploy in real-world where people are tired, they have been working long hours or they are not comfortable with computers and suddenly that benefit drops to 5 percent. (Participant 05)

### What to Evaluate? Selecting the Right Indicators of Quality

Participants indicated that much of the complexity of evaluating CDSS in the healthcare environment owes to varying perceptions among stakeholders. Guidelines on “what” should be measured to evaluate system quality are fragmented. Some participants suggested that indicators of system quality need to be defined at a very local level, thus they can be different across sites with different priorities, while other participants believed that shared baseline quality measures must be established across organizations to evaluate the impact of CDSS on care services.

[The] question is what quality means. There is no standard answer to it because that’s oversimplifying an issue for a very complex system. So, it can be different across different sites with different priorities. (Participant 11)

Another participant explained as follows:

The element of quality metrics must be based on the value judgement of the system by all the stakeholders based on their varying interests and perceptions. The success measures, which are also recognised by your majority stakeholder groups, provide a robust evaluation and feedback process that is transparent for everyone to see and buy into. (Participant 03)

Some participants mentioned that the Australian Digital Health Agency’s benefits evaluation framework marks a positive step in seeking to capture a broad range of outcomes quantitatively and qualitatively. This framework includes varying work streams, such as “impact evaluations,” “behavioral economics” and “customer and market insights” in the evaluation process. It can enable the assessment of CDSS performance from the perspective of different stakeholders and accommodate the relative significance of different outcomes for different stakeholder groups (Box 1, Quotes 1 and 2).

Box 1. Participant quotes illustrating “what” to evaluate

1. Evaluation needs to be multi-dimensional and can operate at different levels. You really need to have a strong argument on why these systems work, not just from a clinical or economic point of view but how they improve the quality and safety of care services. This requires taking a 360° view of a lot of different aspects that are important to different players – patients, clinicians, funders, and governing bodies. (Participant 02)
2. We need evaluation models which consider all kinds of possible relationships between different variables, considering that the value of digital health is created and delivered in an ecosystem comprising different technologies and stakeholders. We need to understand what I might call [the] spatial interaction of the technology with the environment, context, or actors. (Participant 10)
3. Part of the evaluation is validating the system’s logic, but if your system is using artificial intelligence, then it is pretty opaque. We still don’t know how to validate that logic. I don’t think we do it properly in the Australian healthcare system in any shape or form. We are relying on traditional evaluation approaches to deal with this new class of systems. (Participant 01)

While participants frequently recommended a holistic evaluation approach, many participants also believed that, since concepts of cognitive computing and artificial intelligence are gaining attention in CDSS literature, the evaluation methods are unable to match the pace of innovation (Box 1, Quote 3).

### How to Evaluate? Uncertainty about the Process

Participants believed that what needs to be measured and how the process of evaluation is to be designed depends on one another. There was a consensus that the complex infrastructure and resistance to CDSS adoption that are typical of healthcare settings make the evaluation process challenging. Some participants were concerned that evaluation approaches are built upon existing regulations, thus lacking the agility needed to match the speed of innovation in system designs and frequent upgrades. Participants indicated that the time taken by traditional evaluation methods to generate evidence is usually more than the software development and upgrade cycle, meaning that evaluation can never keep up with its moving target (Box 2, Quote 1).

Box 2. Quotes illustrating “how” to evaluate

1. Our current evaluation approaches are unable to match the speed of software development life. For example, randomised controlled trials have been established as a gold standard to generate evidence in the medical literature. But, along with other concerns such as randomisation is unethical in medical informatics, RCTs are too expensive and take too long when technology gets upgraded too quickly. (Participant 05)
2. A large proportion of decision support software or add-ons embedded in the medication management systems falls outside the jurisdiction of TGA regulations. One, their purpose might be to provide information on clinical practice guidelines and clinical studies and, second, healthcare professionals can choose to act upon the information or alert. In such cases, we don’t know how to determine the safety or credibility of these software. (Participant 07)

Some participants believed that evaluation methods are limited to usability testing, and therefore are confined to end-users and post-implementation phases. However, while different stakeholders, such as healthcare providers, vendors, enterprises, and regulators, may share similar challenges in conducting evaluation studies, such as cost and time pressures, they also have specific requirements that suggest it might be more effective to carry out an evaluation at different phases of the CDSS implementation. One participant suggested:

Evaluation can be done better by involving the right people at the right stage to know what actually needs to be evaluated. It is not as longitudinal as it should be, it’s a more cross-sectional analysis style. That’s why all we get out of these evaluations is that your implemented system is not being optimally adopted. But we don’t get answers to what or why different people have concerns about the system. (Participant 05)

Participants indicated that evaluation approaches are usually selected depending upon the software’s intended use. Digital health systems are regulated only if they fulfill the definition of a medical device. However, participants argued that there is no guidance available for determining the quality and safety of CDSS that do not meet this definition (Box 2, Quote 2).

### When to Evaluate?

Participants generally agreed that evaluation must be carried out across the CDSS project’s lifecycle. They believed that evaluation could take different forms and use different approaches depending on the requirements of stakeholders and project phases. One participant specifically explained:

For decision support software, theoretically speaking we have established international standards for evaluation. It provides the quality model with phases such as internal quality assessment for evaluating the software requirements while it is being developed or external quality assessment to test the performance in a simulated setting and then ongoing usability assessment while the system is in use in actual settings. But the problem I have seen in CDSS projects is that the evaluation is not properly funded or designed as an ongoing project phase, it is usually an afterthought and is often done quite badly. (Participant 10)

Many participants believed that extensive use of the pre-post design in evaluation studies of CDSS has helped in assessing CDSS performance. However, pre-post design has also shifted the focus away from evaluation being an ongoing process for continuous optimization in the design and implementation of CDSS (Box 3, Quotes 1 and 2). Participants suggested that with the increasing complexities of digital health systems, there is a need to adopt innovative designs for evidence generation based on stakeholder requirements, outcome measures, and interactions between digital and non-digital aspects of the clinical environment. This was seen as a way to develop an integrative evaluation approach based on real-world evidence.

Box 3. Quotes illustrating “when” to evaluate

1. We tend to focus more on end-point evaluation rather than the before you implement the system, as you are using the system, as you are working with vendors to a point where you are ready to implement the system, as you are implementing it and then after that post-implementation and then as an on-going process. Therefore, we have no way of knowing what works and what doesn’t along the lifecycle of the project and that’s a safety and quality risk. (Participant 01)
2. We are looking for optimisation beyond the implementation. It should not be like here is your system, see ya later. The process of validation should not be limited to once when you introduce the system, but you must do it at regular time points which are planned beforehand with the consultation of all stakeholders. (Participant 06)

## Discussion

Digital health systems such as CDSS are increasingly being used as tools that enable personalized care and the optimization of data flow within and between healthcare organizations. However, there are some challenges in assessing their impact on the care process and ensuring compliance with quality standards. In this paper, we have investigated different factors that can limit timely and robust CDSS evaluation in the Australian healthcare system. Our results have shown that CDSS are usually evaluated as a local technical system, rather than in relation to digital transformation in organizations, behaviors, policies, and the wider healthcare system. The following sections will discuss the implications of our findings for the Australian healthcare system across the CDSS life cycle.

### Premarket Assessment

For regulators, the evidence requirements for evaluating the safety and efficacy of digital health products depend on their intended use (9;20). As such, a significant proportion of CDSS usually fails to meet the definition of a medical device, thus falling outside the jurisdiction of regulatory bodies. Based on technical complexity and clinical risk, different CDSS may have different regulatory considerations. In Australia, the regulation of software-based products is overseen by the Therapeutic Goods Administration (TGA). Currently, CDSS are only subjected to TGA regulation if they meet the legal definition of a medical device, which can be summarized as any technology or software whose intended use is to prevent, diagnose, predict, treat, or monitor disease (21). Like other regulatory agencies, TGA adopts a risk-classification approach to regulate CDSS, so it is important to remember that there is always a certain level of risk of harm to patients if CDSS undergoes changes or provides information that is not accurate. Our findings suggested that several CDSS fitting the definition of a medical device could be exempted from TGA regulation on the basis that they provide recommendations for a diagnosis or treatment and are not intended to replace the clinical judgment of a health professional. It could be argued that all medical devices providing clinical information need integration with the clinical judgment of a health professional, so presumably this exemption decision was made because of an assessment that there was a low risk to the patient. Developers can introduce changes in the prediction algorithms via software upgrades, meaning that CDSS – that previously only provided an advisory function – can transition to providing a specific diagnosis or specifying the treatment plan (7;22). It is uncertain how CDSS exempted by the TGA would be monitored continuously, specifically as the software changes as a part of an upgrade cycle.

Our findings further highlighted that challenges for CDSS market approval extend beyond the assessment of clinical efficacy and safety. They extend to accurately assessing uncertainties associated with models and data, ensuring cybersecurity measures are robust, and assessing the impact of software integration within the complex clinical environment. At present, regulatory frameworks lag behind rapid technological advancement. Indeed, they may always do so in the case of CDSS, since by the time market authorization is granted, the software may already be obsolete. This misalignment may be attributable to the evidentiary requirements of regulatory bodies having been developed for health technologies with well-defined impacts, where the nature of digital health systems such as CDSS make the prediction of precise impacts almost impossible.

The misalignment between the regulatory process and technological advancement may also reflect the different primary interests of technology developers and regulators. As participants in our study indicated, developers primarily want rapid access to market, while regulators primarily want to ensure clinical efficacy and safety. This warrants, not abandoning the robust evaluation, but developing pragmatic and adaptable regulatory strategies that integrate complexities of emerging technologies such as CDSS in a healthcare system characterized by different actors, processes, and practices. Our findings are consistent with the WHO guiding principles of the digital health global strategy 2020–2025, which highlights that digital health regulations should be guided by a robust strategy that integrates technological, clinical, financial, organizational, ethical, and social aspects (23).

### Postmarket Assessment

#### Internal Evaluation

We identified that there is no clear framework in Australia for reimbursing most digital health products and services such as CDSS. Generally, for software that integrates within a device, service, or prosthesis, Australia’s HTA-based committees require evidence that the software represents a clinically effective and cost-effective alternative to current healthcare practices, in order to responsibly steward public resources. Along with clinical and cost-effectiveness, the Medical Services Advisory Committee (MSAC) in Australia considers less-readily quantifiable factors such as equity, the value of knowing, ethical and social concerns, and impact on organization in its decision-making (24). However, there is a limited guidance on what factors should be evaluated for digital health systems.

The applicability of HTA frameworks may differ across public and private organizations. Currently, the implementation of CDSS is mostly decentralized, and limited to privately funded health services. Thus, the evaluation of CDSS is usually carried out at an organizational level with minimal or no involvement of government departments and, thus, potentially with insufficient HTA or health services research expertise. We further found that due to limited resources and HTA capability in small privately funded organizations, the evaluation process may lack the rigor required to sufficiently assess the risks and benefits associated with CDSS. Most organizations assess CDSS effectiveness using disparate quality measures such as improvement in clinical processes, system adoption, and acceptance. These are unable to account for the interaction of CDSS with organizational processes, the clinical culture, individual practices, and value for the health system overall.

Many of our participants believed that CDSS needs to be assessed within a specific organizational context, because different organizations have different CDSS implementation plans and timelines. Considering the organizational context and trajectories of change can help one to understand how the same system may have varying effects in different teams, units, and organizations. The relationship between digital health systems and their environment is usually non-linear (25). However, many existing evaluation approaches assume linear causation and are unable to reflect the complexity of different clinical environments. In practice, a dynamic environment and an evolving technology can give rise to variability in CDSS performance and organizational response. Therefore, considering multiple factors (such as modifications to clinical workflows, communications, end-users’ skills and training, and clinical culture) can help to ensure that CDSS impact is properly assessed.

Due to the observed disparate evaluation processes in Australia, there is a need for a national evaluation framework for digital systems with higher involvement of government HTA-based committees to establish the value of CDSS for the broader healthcare system.

#### Continuous Monitoring

CDSS upgrades (such as changes in system performance, new or modified functions, and new indications for use) can change the software’s effectiveness or safety. From a patient care perspective, continuous evaluation should focus on the potential risks or change in the level of an existing risk to patient safety that might result from upgrades. A recent study has suggested that upgrades and post-market software changes must be part of the monitoring framework (26). The FDA has recognized that existing evaluation frameworks are not well suited to rapidly evolving medical software, thus a Software Precertification Program was introduced in which *software developers* were evaluated for excellence rather than the individual products (27). It is the responsibility of the manufacturers who have achieved pre-certification to continuously monitor their software for effectiveness throughout the product lifecycle while ensuring iterative improvement and maintaining patient safety in the real world. It is hoped that this pre-certification process, combined with post-market surveillance by organizations, will shorten the evaluation timeline without stifling innovation. Achieving an acceptably low level of risk to patient safety requires robust evaluation throughout the CDSS lifecycle and continuous post-market oversight, as seen in the FDA pre-certification program. As discussed above, developers may want to have less regulation and quicker access to market, therefore initiatives like the FDA pre-certification program cannot be implemented in Australia without significant critical oversight from the government (e.g., HTA engagement).

Our findings indicate that evaluations fail to accommodate the rapidly evolving nature of digital technologies potentially because rapid evolution conflicts with traditional evidence-generation approaches (9). Thus, software potentially undergoes significant changes by the time the evaluation study is complete, making the results of the evaluation irrelevant. This is especially important for non-knowledge-based CDSS that changes continuously over time. Some recent studies have proposed using observational or real-world evidence (RWE) to evaluate digital health systems, specifically evaluation using data generated by end-users as they engage with the system (28). This may help manufacturers and governments to assess how CDSS are being used, identify opportunities to improve the product, and quickly address any risk to patient safety. It may also help with assessing the impact of the system on health outcomes in real-time and with understanding any digitally mediated change in clinical practice and behavior.

In this study, we have identified different challenges for evaluating CDSS. Because of our focus on the Australian healthcare system, not all findings may be generalizable to a broader global context. However, many key findings, such as the need for a socio-technical lens in evaluation, processes, and data-driven analytical methodologies do seem important in a global context, because of their appearance in similar studies outside of Australia (14;25;28).

Our findings include pragmatic recommendations provided by interviewees (Table 1) for an effective and successful CDSS evaluation.

**Table 1.** Recommendations for CDSS evaluation

Themes	Recommendations
Why evaluate?	Introducing a CDSS into the care environment can impact other clinical elements in ways that are difficult to predict. Effective and holistic evaluation is needed to understand the interaction of CDSS with organizational processes, the clinical culture, and individual practices
What to evaluate?	Stakeholders differ on what matters most, so some participants believed that indicators of CDSS quality should be defined at a local level, while others believed that some indicators of quality should be established across organizations The CDSS evaluation must accommodate the perspective of different stakeholders and the relative significance of different outcomes for different stakeholder groups
How and when to evaluate?	The rapid software upgrade cycle can render traditional evaluation approaches irrelevant. Therefore, any evaluation process should not be limited to testing usability post-implementation, but should be continuous, spanning the entire CDSS lifecycle and taking advantage of the generation of real-world evidence

CDSS, clinical decision support software.

## Conclusion

As innovation and diversity in CDSS increase, so too does the need for systematic and continuous evaluation to ensure that clinical recommendations provided by the software are safe and effective in improving the quality of care. Our research found that quality measures based on clinical outcomes and usability have overly narrowed the scope of evaluation and failed to consider the broader impact of the sociotechnical environment on CDSS performance. The existing evaluation methods have inherent limitations since they attempt to establish linear causation when CDSS operate within a complex clinical environment. To account for this complexity in the evaluation process, a living HTA that is continuously updated with real-world evidence may provide a better understanding of CDSS impact, particularly if attention is given to the interaction between technical, environmental, social, and behavioral factors. The lack of post-market monitoring of CDSS may have serious consequences for patient safety, particularly when developers, who are mostly profit-driven and do not have the same duty of care that governments do, introduce unmonitored changes in the software as a part of a software upgrade.

## Supporting information

Laka et al. supplementary material Laka et al. supplementary material

## Supplementary material

The supplementary material for this article can be found at https://doi.org/10.1017/S0266462324000059.

## References

[1] Merlin T, Street J, Carter D, Haji Ali Afzali H. Challenges in the evaluation of emerging highly specialised technologies: Is there a role for living HTA? Appl Health Econ Health Policy. 2023;21:823–830. 37824056 10.1007/s40258-023-00835-3 PMC10628011

[2] Sutton RT, Pincock D, Baumgart DC, et al. An overview of clinical decision support systems: Benefits, risks, and strategies for success. NPJ Digit Med. 2020;3:17. 32047862 10.1038/s41746-020-0221-y PMC7005290

[3] Shortliffe EH, Sepúlveda MJ. Clinical decision support in the era of artificial intelligence. JAMA. 2018;320:2199–2200. 30398550 10.1001/jama.2018.17163

[4] Musen MA, Middleton B, Greenes RA. Clinical decision-support systems. Biomedical Informatics. Cham: Springer; 2014. p. 643–674.

[5] Pawloski PA, Brooks GA, Nielsen ME, Olson-Bullis BA. A systematic review of clinical decision support systems for clinical oncology practice. J Natl Compr Cancer Netw. 2019;17:331–338. 10.6004/jnccn.2018.7104 PMC6563614 30959468

[6] Ammenwerth E, Gräber S, Herrmann G, Bürkle T, König J. Evaluation of health information systems—Problems and challenges. Int J Med Inform. 2003;71:125–135. 14519405 10.1016/s1386-5056(03)00131-x

[7] Lobach DF Evaluation of clinical decision support. In: Berner ES, editor. Clinical decision support systems: Theory and practice. Cham: Springer International Publishing; 2016. p. 147–161.

[8] Magrabi F, Ammenwerth E, McNair JB, et al. Artificial intelligence in clinical decision support: Challenges for evaluating AI and practical implications. Yearb Med Inform. 2019;28:128–134. 31022752 10.1055/s-0039-1677903 PMC6697499

[9] Guo C, Ashrafian H, Ghafur S, et al. Challenges for the evaluation of digital health solutions—A call for innovative evidence generation approaches. NPJ Digit Med. 2020;3:110. 32904379 10.1038/s41746-020-00314-2 PMC7453198

[10] Murray E, Hekler EB, Andersson G, et al. Evaluating digital health interventions: Key questions and approaches. Am J Prev Med. 2016;51:843–851. 27745684 10.1016/j.amepre.2016.06.008 PMC5324832

[11] Mathews SC, McShea MJ, Hanley CL, et al. Digital health: A path to validation. NPJ Digit Med. 2019;2:38. 31304384 10.1038/s41746-019-0111-3 PMC6550273

[12] Eslami Andargoli A, Scheepers H, Rajendran D, Sohal A. Health information systems evaluation frameworks: A systematic review. Int J Med Inform. 2017;97:195–209. 27919378 10.1016/j.ijmedinf.2016.10.008

[13] Laka M, Milazzo A, Merlin T. Can evidence-based decision support tools transform antibiotic management? A systematic review and meta-analyses. J Antimicrob Chemother. 2020;75:1099–1111. 31960021 10.1093/jac/dkz543

[14] Enam A, Torres-Bonilla J, Eriksson H. Evidence-based evaluation of eHealth interventions: Systematic literature review. J Med Internet Res. 2018;20:e10971. 30470678 10.2196/10971 PMC6286426

[15] Laka M, Carter D, Milazzo A, Merlin T. Challenges and opportunities in implementing clinical decision support systems (CDSS) at scale: Interviews with Australian policymakers. Health Policy Technol. 2022;11:100652.

[16] Stockdale R, Standing C. An interpretive approach to evaluating information systems: A content, context, process framework. Eur J Oper Res. 2006;173:1090–1102.

[17] Sockolow P, Crawford P, Lehmann H. Health services research evaluation principles. Methods Inf Med. 2012;51:122–130. 22311125 10.3414/ME10-01-0066

[18] Boyatzis RE (1998) Transforming qualitative information: Thematic analysis and code development. Thousand Oaks, CA: Sage.

[19] Crabtree BF, Miller WF. A template approach to text analysis: Developing and using codebooks. In: Crabtree BF, Miller WF, editors. Doing qualitative research. Newbury Park, CA: Sage; 1999. p. 163–177.

[20] Moshi MR, Parsons J, Tooher R, Merlin T. Evaluation of mobile health applications: Is regulatory policy up to the challenge? Int J Technol Assess Health Care. 2019;35:351–360. 31307566 10.1017/S0266462319000461

[21] Therapeutic Goods Administration. Clinical decision support software: Scope and examples; 2021.

[22] Biggs JS, Willcocks A, Burger M, Makeham MA. Digital health benefits evaluation frameworks: Building the evidence to support Australia’s National Digital Health Strategy. Med J Aust. 2019;210:S9–S11. 30927475 10.5694/mja2.50034

[23] World Health Organization (WHO). Global strategy on digital health 2020–2025. Geneva; 2021.

[24] Medical Services Advisory Committee. Guidelines for preparing assessments for the Medical Services Advisory Committee; 2021.

[25] Greenhalgh T, Russell J. Why do evaluations of eHealth programs fail? An alternative set of guiding principles. PLoS Med. 2010;7:e1000360. 21072245 10.1371/journal.pmed.1000360 PMC2970573

[26] Moshi MR, Tooher R, Merlin T. Development of a health technology assessment module for evaluating mobile medical applications. Int J Technol Assess Health Care. 2020;36:252–261. 32419676 10.1017/S0266462320000288

[27] Lee TT, Kesselheim AS. US Food and Drug Administration precertification pilot program for digital health software: Weighing the benefits and risks. Ann Intern Med. 2018;168:730–732. 29632953 10.7326/M17-2715

[28] Pham Q, Shaw J, Morita PP, et al. The Service of Research Analytics to optimize digital health evidence generation: Multilevel case study. J Med Internet Res. 2019;21:e14849. 31710296 10.2196/14849 PMC6878108

