# Supplementary material for: Evaluating clinical decision support software (CDSS): challenges for robust evidence generation
Source: Int J Technol Assess Health Care. 2024 Feb 8;40(1):e16. doi: 10.1017/S0266462324000059 (PMC11570080; doi:10.1017/S0266462324000059)
Supplement: Laka et al. supplementary material [file S0266462324000059sup001.docx]

**Interview Schedule**

**Only a subset of questions related to the evaluation of CDSS is provided below:

**Question 1:**

Do you think it is important to evaluate the CDSS performance? If yes, why?

**Follow-up:**

- *Can evaluation play any role in identifying any potential risk to patient safety? If yes, can it also help in mitigating that risk?*
- *Can the evaluation of CDSS also impact the quality of care?*

**Question 2:**

How can the quality of CDSS be defined and established?

**Follow-up:**

- *Can it differ across organisations? If yes, why?*
- *Can it have a different meaning for different stakeholders?*

**Question 3:**

What are the important aspects that must be considered for an effective evaluation of CDSS?

**Follow-up:**

- *How is an evaluation approach selected? What factors are considered in the selection of a specific approach?*
- *Do fluidity of the digital technology play any role in an evaluation approach?*
- *Changing roles of actors/stakeholders in healthcare*

**Question 4:**

In your experience, what do you see as major challenges in evaluating CDSS?

**Follow-up:**

- *What are the challenges specific to Australia?*
- *Do you think that these challenges are unique to digital health technologies? Do these challenges differ from other medical devices?*

**Question 5:**

To ensure the quality, safety, and efficacy of devices, drugs or products with medical or health-associated uses, there are multiple regulatory frameworks. Do you think there is a specific regulation framework available for digital health systems such as CDSS?

**Follow-up:**

- *If yes, how effective it is in evaluating CDSS?*
- *If not, why do you think it is not effective? What can we do about this?*
- *Can the performance and safety of CDSS be established before implementing in real-world settings?*
- *How often should CDSS be evaluated along the product life cycle?*

**Question 6:**

In your experience, how are CDSS currently regulated in Australia?

**Follow-up:**

- *Do you see any issues or shortcomings in the current regulations of CDSS in Australia?*
- *What kind of evidence is required?*
- *Do you have any recommendations for CDSS evaluation?*
